# Diversity of the Neglected and Underutilized Crop Species of Importance in Benin

**DOI:** 10.1100/2012/932947

**Published:** 2012-04-19

**Authors:** A. Dansi, R. Vodouhè, P. Azokpota, H. Yedomonhan, P. Assogba, A. Adjatin, Y. L. Loko, I. Dossou-Aminon, K. Akpagana

**Affiliations:** ^1^Laboratory of Agricultural Biodiversity and Tropical Plant breeding (LAAPT), Faculty of Sciences and Technology (FAST), University of Abomey-Calavi, BP 526, Cotonou, Benin; ^2^Crop, Aromatic and Medicinal Plant Biodiversity Research and Development Institute (IRDCAM), 071BP28, Cotonou, Benin; ^3^Bioversity International, Office of West and Central Africa, 08 BP 0931, Cotonou, Benin; ^4^Department of Nutrition and Food Technology, Faculty of Agriculture (FSA), University of Abomey-Calavi, BP 526, Cotonou, Benin; ^5^National Herbarium, Department of Botany and Plant Biology, Faculty of Sciences and Technology (FAST), University of Abomey-Calavi (UAC), BP 526, Cotonou, Benin; ^6^Laboratoire de Botanique, Faculté des sciences (FS), Université de Lomé, BP 1515, Lomé, Togo

## Abstract

Many of the plant species that are cultivated for food across the world are neglected and underutilized. To assess their diversity in Benin and identify the priority species and establish their research needs, a survey was conducted in 50 villages distributed throughout the country. The study revealed 41 neglected and underutilized crop species (NUCS) among which 19 were identified as of priority base on 10 criteria among which included their extent and degree of consumption. Reasons for neglect vary with the producers and the agricultural technicians. Market surveys revealed that NUCS are important source of household incomes and substantially contribute to poverty reduction. Review of the literature available revealed that most of the species are rich in nutrients and have some proven medicinal values and the promotion of their use would help in combating malnutrition and improving the health status of the local populations. The knowledge gaps and research needs are immense on most of the species identified as no concrete scientific data is nationally available. In terms of research, almost all has to be done starting from basic ethnobotanical investigation. The results will help the scientists and students willing to conduct research on NUCS in Benin to better orient their research programs.

## 1. Introduction

Across the world, many of the plant species that are cultivated for food are neglected and underutilized while they play a crucial role in the food security, nutrition, and income generation of the rural poor [1]. While these crops continue to be maintained by cultural preferences and traditional practices, they remain inadequately characterised and neglected by research and conservation. Lack of attention has meant that their potential value is underestimated and underexploited. It also places them in danger of continued genetic erosion and disappearance which would further restrict development options for the poor. Many neglected and underutilized crop species (NUCS) are nutritionally rich [2–4] therefore, their erosion can have immediate consequences on the nutritional status and food security of the poor and their enhanced use can bring about better nutrition and fight hidden hunger. A lot of NUCS are recorded to be adapted to difficult environments unfit for other crops where they can provide sustainable productions [5]. In this way, they contribute significantly to maintain diversity rich and hence more stable agroecosystems. Little is known about the ecology of the NUCS or how to improve varieties' yield and quality, and little has been done to identify the most effective commercialization, marketing, and policy frameworks to promote their use and maximize their economic value [6]. With regard to all these, conservation of, and research on, these NUCS is needed for better maintenance of their resource base, to ensure their development and their sustainable use by present and future generations.

For the promotion of NUCS to be a reality in Africa, a large cadre of well-trained and motivated African agricultural scientists will have to play a critical role in providing farmers with a steady flow of new technologies (improved farming practices, newly developed varieties, etc.). Unfortunately, because of decades of underinvestment mainly in African regions, the human and institutional capacity required for research, marketing, and knowledge sharing on NUCS is weak or absent. To address this issue, RUFORUM (Regional Universities Forum for Capacity Building in Agriculture) in collaboration with Bioversity International and four universities/research institutes located in Benin, Ghana, Kenya, and Malawi has launched in 2010 a new project entitled building human and institutional capacity for enhancing the conservation and use of Neglected and Underutilized Species of crops in West Africa, and Eastern and Southern Africa. In this project, national fact finding studies on the diversity of the Neglected and Underutilized Species (NUS) of food crops was planned for four countries including Benin, Ghana, Kenya, and Malawi. We report in this paper the results of the study conducted in Republic of Benin in order to identify and prioritize the countries' neglected and underutilized crop species of importance and document the knowledge gaps associated with them that can be addressed by both national and international communities.

## 2. Methodology

### 2.1. The Study Area

The study was conducted in the Republic of Benin situated in West Africa between the latitudes 6°10′N and 12°25′N and longitudes 0°45′E and 3°55′E [7]. The country covers a total land area of 112,622 km^2^ with a population estimated at about 7 million Inhabitants [8]. It is partitioned into 12 departments inhabited by 29 ethnic groups [7]. The south and the centre are relatively humid agroecological zones with two rainy seasons and mean annual rainfall varying from 1,100 to 1,400 mm/year [7]. The north is situated in arid and semiarid agro-ecological zones characterized by unpredictable and irregular rainfall oscillating between 800 and 950 mm/year with only one rainy season. Mean annual temperatures range from 26 to 28°C and may exceptionally reach 35–40°C in the far northern localities [8, 9]. The country has over 2,807 plant species [9]. Vegetation types are semideciduous forest (South), woodland and savannah woodland (Centre-East and Northeast), dry semideciduous forest (Centre-West and south northwest), and tree and shrub savannahs (far north) [9].

### 2.2. Villages and Institutions Surveyed

**Figure 1:**
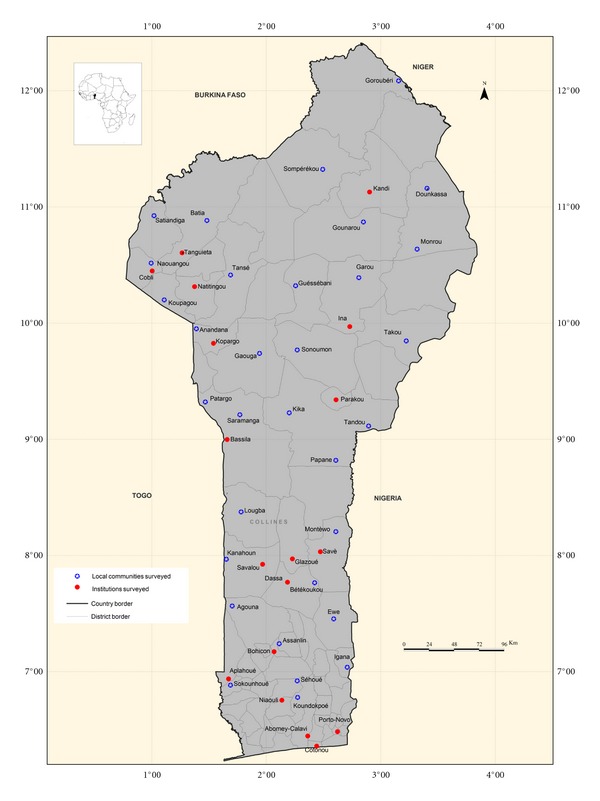
Benin map showing the geographical location of the villages and institutions surveyed.

For the country to be sufficiently covered and for an exhaustive species inventory, 50 villages (Table 1; Figure 1) located in diverse agroecological (humid, semiarid, and arid) and ethnic zones were randomly selected for the study.

In Republic of Benin, the agricultural research is globally managed by INRAB (Institut National des Recherches Agricoles du Benin), IRDCAM (Institut de Recherche et de Développement sur la Biodiversité des Plantes Cultivées, Aromatiques et Médicinales), and the national universities (Parakou and Abomey-Calavi) while the popularisation is mainly the prerogative of the Ministry of Agriculture and its divisions. INRAB has four Agricultural Research Centres (CRA) working on subsistence crops and located at Agonkanmè (CRA-Agonkanmè), Niaouli (CRA-Sud), Savè (CRA-Centre), and Ina (CRA-Nord) (Figure 1). It also has a special vegetable programme (PCM) based at Agonkanmè and a Research and Development team (RD) based at Natitingou. The Ministry of Agriculture has a principal division (CeRPA) in all the departments and a subdivision (CeCPA) in all the districts. In addition, the Ministry of Agriculture also has a Department of Agriculture (DAGRI) located at Porto-Novo and a national society for the promotion of agricultural products (SONAPRA) located at Parakou. The two universities through their Faculties of Agriculture and the Faculty of Sciences and Technology (FAST) of the University of Abomey-Calavi through its Laboratory of Agricultural Biodiversity and Tropical Plant breeding (LAAPT) and Laboratory of Genetic and Biotechnology, highly contribute to capacity building in crop science. All these delocalised institutions were also selected for the study. In addition the associations of the producers of the districts of Savalou, Aplahoué, Cobli, and Kandi (Figure 1) were also surveyed. Also, the associations of the market women of the most important national agricultural markets (Glazoué, Bohicon, Parakou, and Tanguiéta) were considered as commercialisation of agricultural products in Benin is most exclusively a female enterprise. At total 50 villages, 17 research and development institutions and 11 local farmers and market women organizations were surveyed (Table 1; Figure 1).

### 2.3. Data Collection and Analysis

Data were collected during expeditions from the different sites and institutions through the application of Participatory Research Appraisal tools and techniques such as direct observation, individual interviews, and field visits using a questionnaire [10, 11]. Ten randomly selected individuals were interviewed per village and per institution. In the villages, interviews were conducted with the help of translators from each area and selected farmers were requested in advance to bring samples of the neglected and underutilised crops they produce or knew about. Through discussion, some key information was recorded on each of the species identified. These are local vernacular names, type of plant, part used, and period of availability of the part used. Each species was evaluated for ten parameters (extend of the production, extent of consumption, degree of consumption, perceived nutritional value, cultural importance, medicinal properties, market use, market value, contribution to household income, and contribution to women empowerment) using three scores: 3 (low/restricted), 5 (average/regionwide), and 7 (High/countrywide). Field (home gardens, cultivated fields) visits were conducted to see some of the species under cultivation. Scientific names were determined using the Analytic Flora of Benin [9] and pictures were taken for report. Internet and library research (across national research and development institutions) were also conducted for better documentation of the NUCS recorded.

Data were analysed through descriptive statistics (frequencies, percentages, means, etc.) to generate summaries and tables at different levels. To prioritise the NUCS inventoried, a three-step procedure was used. In the first step (step 1) and with the scoring data, species that received high mean value (6 to 7) for at least one criterion were considered as important NUCS. In the second time (step 2), important NUCS that have been already taking into account by specific research projects were removed to obtain national priority NUCS. Afterwards (step 3), identified priority NUCS were ranked within their crop category (cereals, root, and tuber crop, etc.) base on their recorded mean scores. The four-square analysis method [11] was used to classify the identified priority species based on their extent and degree of consumption. In addition, multivariate analysis was conducted using principal component analysis (PCA) with Minitab statistic program (Minitab version 14, Minitab Inc., State College, PA, USA). This PCA analysis was based on the priority species identified and the four variables most frequently used by the interviewees to spontaneously appreciate the species and which are extent of production, degree of consumption, extent of consumption, and market value. Also, using the mean values obtained by the species for each of these four variables, pairwise distances between landraces were computed by the NTSYS-pc 2.2 software package [12], using the simple matching coefficient of similarity [13] and a dendrogram was created by Unweighted Pair-Group Method with Arithmetic Average (UPGMA) cluster analysis [14, 15].

## 3. Results and Discussion

### 3.1. Diversity and Prioritisation of the Neglected and Underutilised Crops Species in Benin

**Figure 2:**
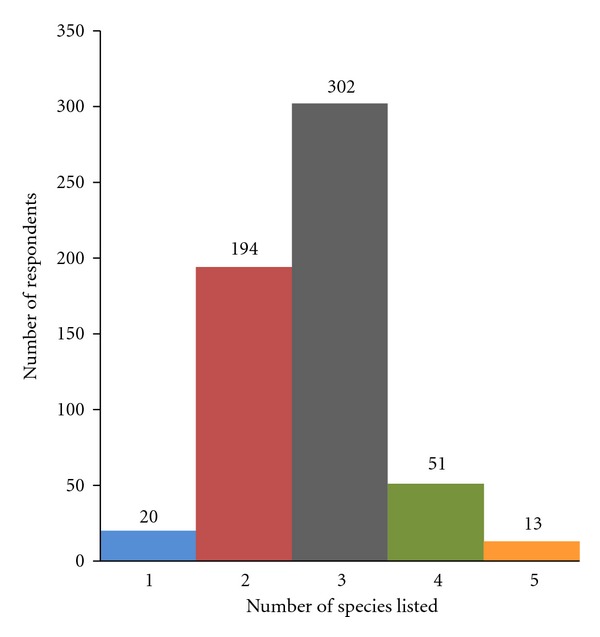
Variation of the number of respondents with the number of species listed.

The study revealed that no document, publication, or project has developed list of neglected and underutilized crop species for Benin. Based on the information gathered from the 580 interviewees and taking into account the types of species excluded by the project (pure medicinal species, spices, and oil crops), 41 crop species were listed as neglected and underutilized in Benin (Table 2). Among these were 3 cereals, 4 roots and tubers, 5 pulses, 13 leafy vegetables, 4 seeds vegetables, and 12 fruits (Table 2). The number of species listed per interviewee varies from 1 to 5 (Figure 2). Most of the interviewees (85.51%) listed 2 to 3 species (Figure 2). Out of the 41 species recorded, 15 (Table 4) received only low to average values for the 10 parameters listed above and 27 (marked with an asterisk in Table 2) were scored high (on average) for at least one of the parameters and were therefore considered as the important neglected and underutilised crops of Benin. The vernacular names, the types of plant, the part used, the period of availability of the important species are summarized in Table 3. From the important species, ten,* Citrullus lanatus, Corchorus olitorius, Crassocephalum rubens, Crassocephalum crepidioides, Cucumeropsis mannii, Ipomea batatas, Launaea taraxacifolia, Macrotyloma geocarpum, Pennisetum glaucum,* and* Vigna subterranea *appear as the most wildly known species as they have been each listed by more than 50% of the interviewees (Table 3).

**Figure 3:**
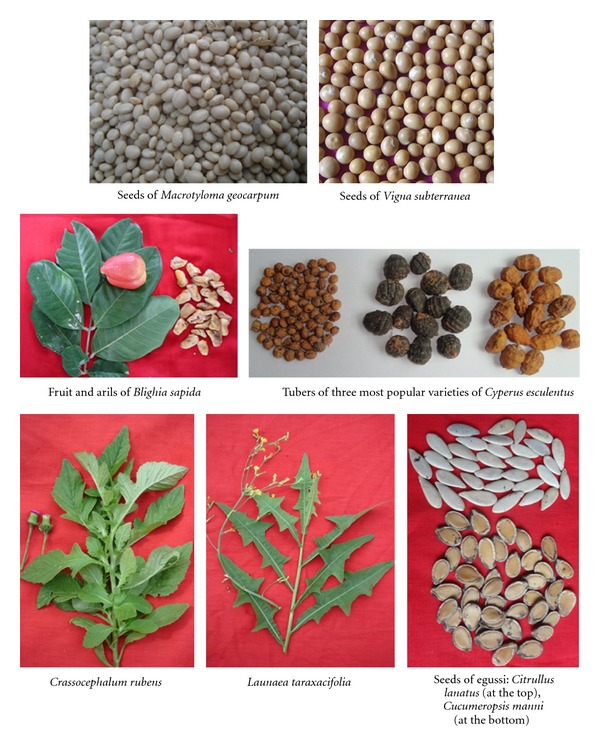
Pictures of selected neglected and underutilized crop species in Benin.

From these 27 species, eight have been already researched on, or considered for research, by specific projects. These were *Acmella oleracea *[16–18],* Adansonia digitata *[19–22],* Digitaria exilis* [11, 23, 24], *Irvingia gabonensis *(newly launched project),* Justicia tenella *[16–18],* Parkia biglobosa *[25, 26], *Sesamum radiatum *[16–18], and *Sorghum bicolor* [27–29]. Removing them from the important species as described above led to 19 species (Table 3) considered as the Benin priority neglected and underutilised crop Species. The priority species were classified within their crop categories and prioritised based on their average score values (Table 2) as follows (species listed by order of importance): cereals (*Pennisetum glaucum*), roots and tubers (*Dioscorea dumetorum *and* Ipomea batatas*), pulses (*Macrotyloma geocarpum, Vigna subterranea, Cajanus cajan, Sphenostylis stenocarpa*), leafy vegetables (*Corchorus olitorius, Crassocephalum crepidioides, Crassocephalum rubens, Launaea taraxacifolia, Bidens pilosa, Cleome gynandra *and* Ceratotheca sesamoides*), seed vegetables (*Citrullus lanatus, Cucumeropsis mannii *and* Sesamum indicum*), and fruit crop (*Blighia sapida*). Pictures of selected NUCS are presented in Figure 3.

**Figure 4:**
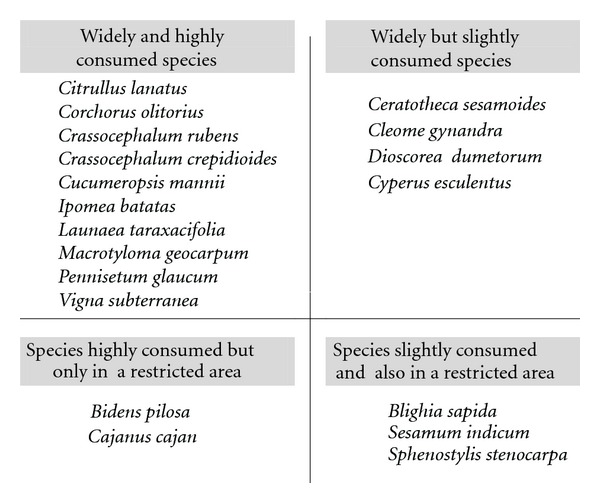
Classification of the Benin priority neglected and underutilized crop species according to their extent and degree of consumption.

**Figure 5:**
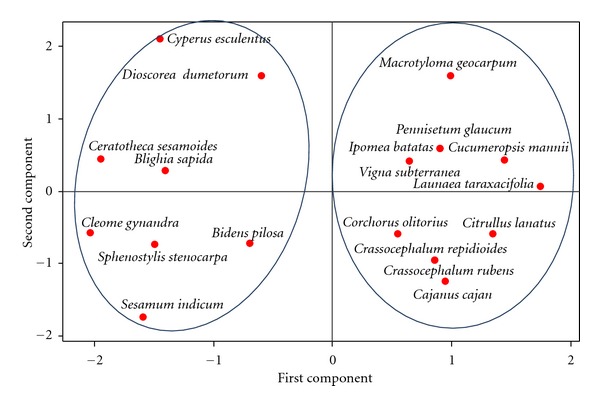
Principal component analysis showing different groups within the priority NUCS.

**Figure 6:**
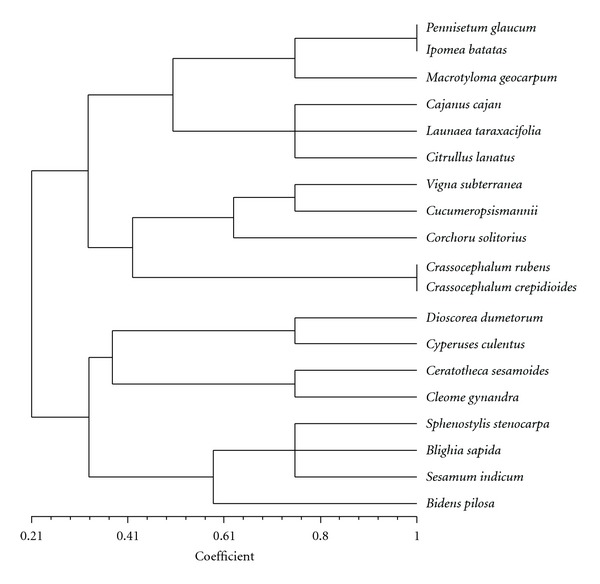
UPGMA dendrogram showing different grouping of the species based on the four most used evaluation criteria.

The four-square analysis carried out based on the extent and the degree of the consumption of the different priority species (Figure 4) revealed that most of them (10 out of the 18 identified) are widely and highly consumed while 3 species (*Ceratotheca sesamoides, Cleome gynandra, *and* Dioscorea dumetorum*) are widely (regionwide that is, north/south or countrywide) but slightly consumed (Figure 4) and two species (*Bidens pilosa *and* Cajanus cajan*) are highly consumed but only in a restricted area represented by the Adja cultural area in the south east of Benin. Species grouped in the first quadrant are also the ten mostly cited species during the survey (Table 3). The principal component analysis (Figure 5) classified the 19 priority species into two groups among which one gathering 11 species which are nothing more than the 10 most cited species to which *Cajanus cajan,* the eleventh species (Table 3) is added. The dendrogram (Figure 6) obtained with the UPGMA cluster analysis almost confirmed the PCA grouping. These results demonstrate on one hand the validity and the robustness of the four variables used for the categorization and, on the other hand, the existence within the 19 priorities species of a set of 10 to 11 species (*Citrullus lanatus, Corchorus olitorius, Crassocephalum rubens, Crassocephalum crepidiodes, Cucumeropsis mannii, Ipomea batatas, Launaea taraxacifolia, Macrotyloma geocarpum, Pennisetum glaucum, Vigna subterranea, *and* Cajanus cajan*) that can be considered as of top high priority for the interviewees (researchers, agricultural extensionists, producers, and users) and to which the first financial supports should be devoted in case a national program may not be able to consider all of them at once.

### 3.2. Reasons of Negligence of the Species

The reasons behind the current status of the NUCS vary according to the agricultural technicians (researchers and extensionists) and the farmers which are also consumers (Table 4). The great majority (95.88%) of the agricultural technicians highlighted the lack of financial supports for research oriented on the neglected crops that no farmer interviewed listed (Table 4). This is widely justified since most of the institutions that support research in developing countries directly or indirectly specify “major crops” in their grant announcements. In republic of Benin, it has been recently that the government is thinking about diversification of the production. Hitherto, the main focus of the national research fund has always been the major crops such as Cotton, sorghum, cassava, maize, yam, rice, and beans. Both farmers and agricultural technicians listed as important reasons of neglect the lack of a national promotion policy of the neglected crops, the lack of organised markets as it is the case for cotton and maize, the susceptibility to pests and diseases and the lack of improved cultural practices and varieties (Table 4). Contrary to technicians, farmers underline two other important reasons which are low yield and laborious production. The listed reasons well-examined revealed that agronomic constraints are in majority and would result from the lack or insufficiency of scientific research and could justify, as indicated by many interviewees, the economically not profitable production of neglected crop species. For this last reason producers of the young generation interviewed said to be mostly interested in cash crops like Cotton, sorghum, and cassava that can provide them with a lot of money in a short time.

Also, some cultural believes weigh heavily on the production of some species. For many producers, voluntary digging out (most often by the children) of a plant of Kersting's groundnut in a given cultivated field to check maturity of the seeds provokes an immediate drying out of the whole field in two to three days hence leading to the loss of the year in case seeds are immature. Should the opposite occur, you will have to rapidly harvest the entire field and this is, as they said, painful, labour consuming and costly. In the Fè ethnic area (District of Bantè in central Benin), seeds of African yam beans *Sphenostylis stenocarpa* should be cooked only in the fields and never at home. When this happens, it leads to an epidemic of varicella engendered by the divinity Sagbata (God of the earth). Cultivation and consumption of *Launaea taraxacifolia *is proscribed to the adepts of this same divinity throughout the country. Because of all these, the production of many priority NUCS is globally being abandoned with as consequence, the disappearing of local varieties (genetic erosion).

### 3.3. Nutritional Value of the Species

Key data available from the Internet and other sources on the nutritional values of the 19 priority NUCS revealed that most of the species have interesting nutritional value and, if promoted, will surely help to combat malnutrition in Benin. For example, chemical evaluation [41], revealed that 100 g edible portion of dried seeds (part consumed) of *Macrotyloma geocarpum* contain water 9.7 g, energy 1457 kJ (348 kcal), protein 21.3 g, fat 1.1 g, carbohydrate 66.6 g, fibre 5.5 g, Ca 103 mg, P 392 mg, Fe 15.0 mg, zinc (4.42 to 4.92 mg), potassium (235.73 to 341.94 mg), thiamin 0.76 mg, riboflavin 0.19 mg, and niacin 2.3 mg. The same authors [41] reported that the content of essential amino acids per 100 g food is arginine (9.3 g), histidine (2.1 g), and phenylalanine (3.2 g), tryptophan 155 mg, lysine 1280 mg, methionine 267 mg, phenylalanine 1125 mg, threonine 738 mg, valine 1209 mg, leucine 1485 mg, and isoleucine 871 mg. The seed of *M. geocarpum* is therefore a high source of crude protein and very low in crude fat. Arginine, an amino acid for pediatric growth, is the most concentrated amino acid in the seed. Arginine, histidine, and phenylalanine concentrations in the seed are higher than the recommended Food and Agricultural Organisation/World Health Organisation amino acid requirement pattern for the preschool age group [41]. The total essential amino acid content in *M. geocarpum *seed is 42.0%. The lysine/arginine ratio calculated to estimate the seed atherogenic potential is 0.3 showing that *M. geocarpum *seed protein will not exert a hypercholesterolemia effect on the consumer.

Similarly, seed of egussi (*Cucumeropsis mannii*) used as vegetable is also a rich source of protein (31.4%), essential amino acids, fat (52.5%), essential fatty acids, minerals, and vitamins making it a nutritive food for consumers in developing countries, especially in West Africa where it is widely cultivated [42–44]. The fatty acids that it contains in abundance are linoleic (62.42%), oleic (15.90%), palmitic (10.27%), and stearic (10.26%) [44]. The high content of polyunsaturated fatty acids and essential amino acids it contains demonstrates that seed of *Cucumeropsis mannii* has potential health benefits and can be incorporated in many African diets and also used in poultry and animal feed [42–44]. Gbolo (*Crassocephalum* sp.) is one of the key traditional leafy vegetables wildly consumed in Benin [10].

In Benin, two species (*Crassocephalum rubens* and *C. Crepidioides*) are used. According to Yehouenou et al. [45], and Adjatin et al. [46], the nutritional values of these two species are slightly different but of high importance. Adjatin et al. [46] reported that the contents of the leaves in raw protein and in crude lipids expressed in % of dry matter are, respectively, 27.13%, 3.45 for *C. crepidioides*; 26.43% and 2.75% for *C. rubens*. The content of vitamin C for 100 g of fresh leaf is of 9.17 mg for *C. crepidioides* and 3.60 mg for *C. rubens*. The content of ash is of 19.76% and 19.02% for *C. rubens* and *C. crepidioides,* respectively. The contents for sodium (Na), potassium (K), magnesium (Mg), calcium (Ca), iron (Fe), Manganese (Mn), and copper (Cu) are 2129.04 mg, 4469.91 mg, 434.13 mg, 3845.88 mg, 1.6 mg, 8.22 mg, and 2.6 mg, respectively, for *C. rubens* and these are higher than those in *C. crepidioides*.

Tigernut (*Cyperus esculentus*) was reported to be an excellent source of some useful minerals such as iron and calcium which are essential for body growth and development [47]. It is high in dietary fibre content, which could be effective in the treatment and prevention of many diseases including colon cancer, coronary heart diseases, obesity, diabetes, and gastrointestinal disorders. In addition, Tigernut has been demonstrated to be a rich source of quality oil and to contain higher essential amino acids than those proposed in the protein standard by the FAO/WHO for satisfying adult needs [47].

The chemical compositions and the nutritional values of other priority NUCS (*Cajanus cajan, Ceratotheca sesamoides, Corchorus olitorius, Citrullus lanatus, Cleome gynandra, Pennisetum glaucum, Sphenostylis stenocarpa, Vigna subterranea, *and* Sesamum indicum*) compiled in Table 5 shows that Africans in general and Beninese in particular suffer or die of malnutrition in the midst of a great diversity of highly nutritive food crops by ignorance or negligence.

### 3.4. Medicinal Value of the Species

Some neglected and underutilized crop species have many important medicinal properties beside their nutritional value and can be used as nutraceutical. Local populations of the surveyed sites believe that* Crassocephalum sp. *has antibiotic, antihelminthes, anti-inflammatory, antidiabetic, anti-malaria and blood regulation properties and also treats indigestion, liver complaints, colds, intestinal worms, and hepatic insufficiency. Recent chemical analysis [46] of leaves extract of this species revealed quite rightly the presence of tannins, flavonoïds, steroids, mucilage, reducing compounds, coumarins, and the C-heterosis that are recognized as possessing pharmacological properties intervening in the prevention and the treatment of several human pathologies such as hypertension, headaches, breast cancer, burns, inflammations, injuries, liver complaints, infections, and sexually transmitted diseases [48, 49].

Leaf of* Cleome gynandra* consumed as leafy vegetable has anti-inflammatory and lysosomal stability actions [50], potent dose-dependent anticancer activity comparable to that of 5-fluorouracil [51] and free radical scavenging activity [52, 53]. It is also believed to improve eyesight and provide energy [33]. Similarly, *Launaea taraxacifolia *is locally believed (indigenous knowledge) to have, through simple and regular consumption as leafy vegetable, lactogenic, aphrodisiaque, antibiotic, and antimalaria properties, a wonderful blood pressure regulating and haemorrhoids treatment capacity [10]. Recently Obi [54] reported that leaves extract of the species has virucidal potential. Gill et al. [55] reported that *Citrullus lanatus *has good antioxidant, anti-inflammatory, and analgesic potential and may be used as a future food medicine. Surprisingly, tuber of *Dioscorea dumetorum* is said to have antidiabetic properties [56, 57]. The leaves of *Bidens pilosa* highly consumed as leafy vegetable in Adja cultural area in the southwest of Benin are reported by the local population to possess antibacterial, antidysenteric, anti-inflammatory, antimicrobial, antimalarial, diuretic, hepatoprotective, and hypotensive activities with most of them being confirmed by scientific research [58]. Tubers of Tigernut (*Cyperus esculentus*) are said to be aphrodisiac, carminative, diuretic, emmenagogue, stimulant, tonic and have potent antioxidant and anti-inflammatory properties [59]. Tigernut tuber has also been reported to be used in the treatment of flatulence, indigestion, diarrhoea, dysentery, and excessive thirst [59]. Therefore, there is the need for increased utilization and awareness about the health benefits of the use of these species.

### 3.5. Economic Importance of the Species

In Benin, no information or data regarding the economic importance of the priority NUCS identified and particularly their income generation role for the poor is available for use. Market survey revealed that all the species are sold in the markets (Table 6) but their market value are highly variable. According to the women association of Glazoué market (the most important agricultural market of central Benin), the price of one kg of *Macrotyloma geocarpum *varies during the year between 2 $ and 4 $ FCFA (which is about 3 to 5 times the price of rice) while the one of *Crassocephalum rubens *varies between 1 $ to 1.5 $ (Table 6). At Bassila (Figure 1) roundabout also known as one of the numerous egussi (*Cucumeropsis edulis *and* Citrullus lanatus*) selling point in Benin, 10 women interviewed reported to individually sell, on average, 30 bags of 2 $ per day hence making a total business of 18000 $ per month. At the road market of Sèhouè where leafy vegetables are among the dominant products sold and during the raining season, *Launaea taraxacifolia* generates to rural market women about 200 $ per day and approximately 6000 $ per month. At the international market of Dantokpa (Cotonou), *Corchorus olitorius* like the other NUCS, is sold through a well-established network of wholesalers and retailers. Rapid survey of 10 women wholesalers revealed an average of 1000 $ income per month and per individual.

At a Tigernut (*Cyperus esculentus*) wholesaling point of this same market, one of the women wholesalers interviewed on the economic importance of this NUC declared: “*I will never leave Tigernut business for other things. During its period of availability, loincloth sellers cannot compete with me with regard to what I spent on and what I gain from in a short time. Look! Apart from Sunday, I paid *20 $* each day and *80 $* all Saturday for tontine. When in a given week wholesalers from Gabon and Cameroun come to the market for buying Tigernut bags, I can easily realize a net benefit of *400 $* this week. Per day I earn about *80 $* on average and we are 15 at this selling point. Make the calculation yourself and see!*” This statement clearly highlighted the economic importance of the species.

Although these data gathered on some species from some selected markets are not results of sound economic analysis, they clearly indicate that the neglected crop species are important source of household incomes and substantially contribute to poverty reduction mostly in rural areas in Benin. In order to provide policy makers with more detailed, robust, and convincing economic data, it is recommended that a scientifically well-designed socioeconomic study be conducted on the identified species.

### 3.6. Gender Role in the Production and Marketing of the Neglected and Underutilized Crop Species

The gender role in the production, diversity preservation, and use of the priority NUCS has to be documented as no data regarding this aspect is available for use. According to the interviewees, the gender role would mostly vary according to the crops and the regions and sometimes the ethnic areas. According to the data collected and summarised in Table 7, leafy vegetables species are produced most exclusively by women while *Dioscorea dumetorum* is essentially men crop. In the north, the production of the pulses *Vigna subterranea *and* Macrotyloma geocarpum* would be women prerogative while in the south and in the centre their production was said to be under the control of both men and women. For all the species apart from *Pennisetum glaucum, Sesamum indicum, *and* Sphenostylis stenocarpa* in the north, the marketing is almost exclusively a female-dominated enterprise. They gain a lot of money through commercialisation of the priority NUCS and this helps them to improve their position within the society. Species which highly contribute to women's empowerment cited are *Macrotyloma geocarpum, Vigna subterranea, Corchorus olitorius, Citrullus lanatus, Cucumeropsis mannii, *and* Launaea taraxacifolia.* The results obtained globally indicate, and as reported by many authors [60–64] for crop production in general, that female farmers contribute more significantly to the production of the priority NUCS species in Benin than their male counterparts. However, the promotion of sustainable production of these species in Benin would require that the needs of both rural male and female farmers be deeply examined and addressed in a comprehensive and systemic manner.

### 3.7. Current Status of the Research, Knowledge Gaps, and Research Needs on the Priority NUCS in Benin

The surveys revealed that in Benin, no research was conducted on *Sesamum indicum*, *Sphenostylis stenocarpa, *and *Dioscorea dumetorum*. On the five leafy vegetables species which are *Bidens pilosa*, *Cleome gynandra, Crassocephalum rubens, Corchorus olitorius,* and *Launaea taraxacifolia* no specific research was conducted apart from Dansi et al. [10, 65] who reported the diversity of their vernacular names and their utilization (degree and extent of consumption, known medicinal values) in the framework of an ethnobotanical investigation of the traditional leafy vegetables in Benin. The National Agricultural Research Institute collected between 2003 and 2005 some accessions of *Macrotyloma geocarpum*, *Pennisetum glaucum, Vigna subterranea,* and *Ipomea batatas* which are now completely lost without the least report of research activities on them. Contrary to these 12 species, some nonnegligible research efforts were made on the remaining six species. On egussi (*Cucumeropsis mannii* and *Citrullus lanatus*) the seeds quality, viability, system, and conservation are studied, an agronomic evaluation is carried out on the farmers' varieties, the cultural practices understood, and the morphological diversity within the species examined [66, 67]. With Pigeonpea (*Cajanus cajan*) research conducted was related to its use in the control of *Imperata cylindrica* (very aggressive weed) population [68], the capacity of the species in improving soil fertility [69], and to its use in cowpea pest management in intercropping system [70, 71]. On *Ceratotheca sesamoides*, an ethnobotanical and ecogeographical survey was conducted to map its geographical distribution and preselect sites for in situ conservation of its genetic resources [16]. Moreover, the genetic diversity within the species has been also assessed using AFLPs [16] as well as its seed quality [18]. The endogenous agroforestry species Ackee (*Blighia sapida*) seems to be the relatively most studied species. Reported research programs devoted to this species include the variation of its vernacular names and the degree of its consumption as vegetable across ethnic areas [10], the documentation of its indigenous knowledge, traditional management and genetic diversity [72], the characterisation of its seed oil [73], the uses, traditional management, perception of variation and preferences of the fruit traits and its implications for domestication [74], and the assessment of the domestication state of the species using AFLP and microsatellite markers [75]. From the study, it appeared that the species are little investigated. Therefore, for each of them there are some gaps to fill in several research fields. The research needs identified per priority species are of diverse natures, and could be organized in 12 topics (Table 8) suitable for future research projects on priority NUCS in Benin. These are ethnobotanical investigation and documentation of the indigenous knowledge, identification and prioritisation of the production constraints, domestication, agromorphological characterisation and genetic diversity analysis, improvement of the agricultural practices, documentation of the pests and diseases, agronomic (yield, biotic and abiotic stresses) evaluation, assessment of the seeds quality and conservation, analyses of the biochemical composition and assessment of the nutritional values, improvement of postharvest conservation and processing technologies, study of the value chains and assessment of the contribution to household income, and germ plasm collection and conservation. Apart from the documentation of the pests and diseases, the agronomic evaluation, the economic studies and the germplasm collection and conservation which are common to all species, the research topics identified vary from one species to another (Table 8). For example among the leafy vegetables ethnobotanical and ecogeographical survey is required for *Crassocephalum sp. *(among others) while seed quality and conservation studies are needed for *Launaea taraxacifolia* and *Ceratotheca sesamoides* with which farmers reported to have tried in vain to conserve the seeds. These research topics identified per priority species will help the scientists and even the postgraduate students willing to conduct research on neglected crop species to better orient their research programs.

## 4. Conclusion

Republic of Benin has a great diversity of neglected and underutilised crop species. These species have enormous nutritional, medicinal, and economic values and when promoted, could highly contribute to poverty reduction mainly in rural areas, and to the improvement of both nutritional and health status of the local populations. Unfortunately they have never been the priority of the national agricultural research system mainly because of lack of political will and financial supports. For the promotion of these neglected and underutilised crop species in Benin, it will be important to put in place a national and special research and development programme under the joint umbrella of the ministries of agriculture and scientific research sponsored by the government involving all the possible actors including researchers, developers, and producers. This national fact-finding study on the neglected and underutilised crop species is, in our knowledge, the first of its type reported in the subregion and should therefore be extended to other countries not included in the project. Considering that the situations across countries may not be too different, we recommend that, for the next coming decade, national and international research and development funders in the field of agriculture prioritize NUCS grants.

## Figures and Tables

**Table 1:** List of the villages and institutions visited and their geographical localisation.

N°s	Villages	Districts	Longitude	Latitude	Institutions visited
1	Abomey-Calavi	Abomey-Calavi	2.3615	6.44508	IRDCAM; UAC; CeRPA
2	Agouna	Didja	1.70278	7.56389	
3	Anandana	Kopargo	1.38944	9.95111	
4	Aplahoué	Aplahoué	1.65765	6.93745	DAP
5	Assanlin	Za-Kpota	2.11389	7.23944	
6	Bassila	Bassila	1.65814	8.9992	CeCPA, DAP; MWA
7	Batia	Tanguiéta	1.48184	10.8835	
8	Bétékoukou	Dassa	2.42313	7.76388	
9	Bohicon	Bohicon	2.06726	7.17133	CeRPA; MWA
10	Cobli	Cobli	1.01135	10.465	DAP
11	Copargo	Copargo	1.54378	9.83028	
12	Cotonou	Cotonou	2.44067	6.35416	MWA
13	Dassa	Dassa	2.18916	7.77049	CeCPA
14	Dounkassa	Kalalé	3.40423	11.1601	
15	Ewe	Kétou	2.58944	7.45361	
16	Gaouga	Djougou	1.94179	9.73933	
17	Garou	Malanville	2.8087	10.3908	
18	Glazoué	Glazoué	2.23818	7.97282	MWA
19	Goroubéri	Karimama	3.15593	12.0852	
20	Gounarou	Gogounou	2.84861	10.8706	
21	Guéssébani	Sinendé	2.25679	10.321	
22	Igana	Pobè	2.70694	7.03667	
23	Ina	N'Dali	2.72929	9.96955	INRAB-CRA
24	Kanahoun	Savalou	1.65179	7.96703	
25	Kandi	Kandi	2.93636	11.1309	DAP
26	Kika	Parakou	2.201	9.2271	
27	Koundokpoé	Zè	2.27389	6.7775	
28	Koupagou	Boukoumbé	1.10886	10.1996	
29	Lougba	Bantè	1.78063	8.37436	
30	Monrou	Segbana	3.3181	10.6369	
31	Montèwo	Savè	2.60746	8.20403	
32	Naouangou	Cobli	0.994473	10.5166	
33	Natitingou	Natitingou	1.37356	10.3131	CeRPA
34	Niaouli	Allada	2.13694	6.7525	INRAB-CRA
35	Papane	Tchaourou	2.60824	8.8185	
36	Parakou	Parakou	2.61002	9.33894	UP; CeRPA; SONAPRA; MWA
37	Patargo	Djougou	1.46789	9.32085	CeCPA
38	Porto-Novo	Porto-Novo	2.6306	6.476	CeRPA; DAGRI; DANA
39	Saramanga	Pénéssoulou	1.7696	9.21111	
40	Satiandiga	Matéri	1.01972	10.925	
41	Savalou	Savalou	1.9688	7.92512	DAP
42	Savè	Savè	2.48345	8.03269	INRAB-CRA
43	Séhoué	Toffo	2.27088	6.91959	
44	Sokounhoué	Aplahoué	1.68803	6.88269	
45	Sompérékou	Banikoara	2.49389	11.3236	
46	Sonoumon	NDali	2.27045	9.76846	
47	Takou	Nikki	3.22343	9.84639	
48	Tandou	Tchatchou	2.89431	9.11341	
49	Tanguiéta	Tanguiéta	1.26533	10.6048	MWA
50	Tansé	Kouandé	1.6887	10.4136	

INRAB: National Agricultural Research Institute; CRA: Agricultural Research Centre; IRDCAM: Crop Aromatic and Medicinal Plant biodiversity Research and Development Institute; CeRPA: Regional Centre for Agricultural Promotion; CeCPA: District Centre for Agricultural Promotion; SONAPRA: National society for the promotion of agricultural products; DAGRI: Department of Agriculture of the Ministry of Agriculture, Livestock and Fishing; DANA: Department of Food and Nutrition of the Ministry of Agriculture, Livestock and Fishing; DAP District Association of Producers; UAC: University of Abomey-Calavi; MWA: Market Women Association; UP: University of Parakou.

**Table 2:** Crop category, evaluation and prioritization of the neglected and underutilized crop species in Benin.

Species	EOC	DOC	EOP	NV	MV	CHI	ADY	CWE	MAV	MU	Average values
Cereals											
Pennisetum glaucum*	Countrywide	High	Regionwide	High	Low	High	Average	Average	Average	High	5.8
Sorghum bicolor*	Regionwide	High	Restricted	High	Low	High	Low	High	Average	High	5.4
Digitaria exilis*	Restricted	High	Restricted	High	Low	High	Low	High	Average	High	5.2
Root and tuber crops											
Dioscorea dumetorum*	Countrywide	Low	Regionwide	Average	Average	High	Low	High	High	High	5.6
Ipomea batatas*	Countrywide	High	Regionwide	Average	Low	High	Average	High	Average	Average	5.6
Cyperus esculentus*	Countrywide	Low	Restricted	Low	High	Average	Low	High	High	High	5.2
*Colocasia esculenta*	Restricted	Low	Restricted	Average	Low	Low	Low	Low	Low	Low	3.2
Leafy Vegetables											
Launaea taraxacifolia*	Countrywide	High	Countrywide	Average	Average	Average	High	High	Average	High	6.2
Sesamum radiatum*	Regionwide	High	Regionwide	High	High	High	Average	High	High	Average	6.2
Crassocephalum rubens*	Countrywide	Average	Countrywide	High	High	High	High	Average	Low	Average	6
Crassocephalum crepidioides*	Countrywide	Average	Countrywide	High	High	High	High	Average	Low	Average	6
Corchorus olitorius*	Regionwide	Average	Countrywide	Average	Average	High	Average	Average	Average	High	5.6
Justicia tenella*	Regionwide	High	Region wide	High	Average	Average	Average	High	Average	Average	5.6
Acmella oleracea*	Regionwide	Average	Restricted	High	High	Average	Average	Average	Average	Average	5.2
Bidens pilosa*	Restricted	Average	Regionwide	Average	Average	High	Average	Average	Average	Average	5
*Vitex doniana*	Restricted	Average	Region wide	Average	Low	Average	Low	Average	Average	Average	4.4
Ceratotheca sesamoides*	Regionwide	Low	Restricted	Low	Low	Low	High	Low	Average	Average	4
Cleome gynandra*	Regionwide	Low	Restricted	Average	High	Low	Low	Low	Low	Average	4
*Talinum triangulare*	Restricted	Low	Restricted	Low	Low	Average	Low	Low	Average	Low	3.4
*Telfairia occidentalis*	Restricted	Average	Restricted	Low	Low	Average	Low	Low	Low	Low	3.4
Pulses											
Macrotyloma geocarpum*	Countrywide	High	Regionwide	High	Average	High	Average	High	High	High	6.4
Vigna subterranea*	Regionwide	Average	Countrywide	Average	Average	High	Low	High	High	High	5.8
Cajanus cajan*	Restricted	High	Countrywide	Average	Low	Average	Average	Average	Average	High	5.2
Sphenostylis stenocarpa*	Restricted	Low	Regionwide	High	Average	Average	Average	Average	Average	Average	4.8
*Phaseolus lunatus*	Restricted	Low	Restricted	Low	Low	Low	Low	Low	Low	Low	3
Seed vegetables											
Citrullus lanatus*	Regionwide	High	Countrywide	High	Low	High	High	High	Average	High	6.2
Cucumeropsis mannii*	Regionwide	High	Countrywide	High	Low	Low	High	High	High	High	6
Parkia biglobosa*	Countrywide	Average	Regionwide	High	Average	Average	Average	High	High	High	6
Sesamum indicum*	Restricted	Low	Regionwide	Low	Low	High	Average	High	Low	Average	4.4
Fruits											
Adansonia digitata*	Regionwide	High	Regionwide	High	High	Average	High	Average	Average	Average	5.8
Irvingia gabonensis*	Regionwide	Average	Regionwide	Average	Low	Average	Low	Average	High	Average	4.8
*Tamarindus indica*	Regionwide	Low	Regionwide	Average	Average	Average	Average	Low	Average	Average	4.6
Blighia sapida*	Restricted	Low	Regionwide	Low	Low	Low	Low	Average	High	Average	4
*Borassus aethiopum*	Restricted	Low	Restricted	Average	Low	Average	Low	Average	Average	Low	3.8
*Chrysophyllum albidum*	Regionwide	Average	Regionwide	Low	Low	Low	Low	Average	Low	Low	3.8
*Uvaria chamae*	Regionwide	Low	Regionwide	Average	Low	Low	Average	Low	Low	Low	3.8
*Ximenia americana*	Regionwide	Low	Regionwide	Average	Low	Low	Low	Low	Low	Low	3.6
*Dialium guineense*	Restricted	Low	Restricted	Low	Low	Low	Low	Low	Average	Average	3.4
*Synsepalum dulcificum*	Regionwide	Low	Restricted	Average	Low	Low	Low	Low	Low	Low	3.4
*Sclerocarya birrea*	Restricted	Low	Restricted	Average	Low	Low	Low	Low	Low	Low	3.2
*Artocarpus altilis*	Restricted	Low	Restricted	Low	Low	Low	Low	Low	Low	Low	3

EOC: extent of consumption; DOC: degree of consumption; EOP: extent of the production; NV: nutritional value; MV: medicinal value; CHI: contribution to household income; ADY: availability during the year; CWE: contribution to women empowerment; MAV: market value; MU: market use.

**Table 3:** List and vernacular names of the neglected and underutilized crop species in Benin.

N°	Species names	Vernacular names	TP	PU	PA	PR (%)
1	*Macrotyloma geocarpum* (Harms) Maréchal & Baudet	Doyiwé (Fon); Nadu (Yoruba, Nago)	H	S	AS	86.37
2	*Vigna subterranean* (L.) Verdc.	Azingokwin (Fon); Ekpa boro (Yoruba, nago); dènsi bontè (Dindi)	H	S	AS	73.79
3	*Crassocephalum rubens* (Juss. ex Jacq.) S. Moore var. *rubens *	Adjèlè (Fè); Akôgbo (Cotafon, Fon, Mahi); Bolo (Adja); Gbolo (Holly, Idasha, Tchabè, Yorouba); Hôhounhôgui (Mahi); Olongôbiè (Ani); Tignikoroya (Wama)	H	L	RS	70.34
4	*Crassocephalum crepidioides *(Benth.) S. Moore	Adjèlè (Fè); Akôgbo (Cotafon, Fon, Mahi); Bolo (Adja); Gbolo (Holly, Idasha, Tchabè, Yorouba); Hôhounhôgui (Mahi); Olongôbiè (Ani); Tignikoroya (Wama)	H	L	RS	70.17
5	*Pennisetum glaucum* (L.) R. Br.	Likun (fon); Emeyè, máyi (Yoruba); Haanibii, Somènè (Dendi)	H	S	AS	66.37
6	*Cucumeropsis mannii* Naud. (Cult)	Goussi-tchègba (fon); Itoò (Yoruba, Nago)	H	S	AS	65.17
7	*Citrullus lanatus *(Thunb.) Matsum. & Nakai	Goussi-gaga (Fon), Itoò (Yoruba, Nago)	H	S	AS	64.65
8	*Launaea taraxacifolia *(Willd.) Amin ex C. Jeffrey	Awonto (Cotafon; Watchi); Gnanri (Holly, Yorouba); Gnantoto (Fon, Mahi); Katakpa (Tchabè); Lôto (Péda; Xwla); Ôdôdô (Fè, Idatcha, Idasha); Wonto, Lanto (Saxwè); Wountou (Adja)	H	L	RS	62.93
9	*Corchorus olitorius *L.	Nainnouwi (Fon, Mahi); Dèmain (Adja); Yôyô (Ani, Bariba, Dendi, Nago, Kotokoli, Lokpa, Natimba, Yom, Wama); Fouam (Berba), Tifanhanti (Ditamari)	H	L	AS	60.17
10	*Ipomoea batatas* (L.) Lam.	Dokouin (Fon, Mahi); Wèli (Mina)	H	T	AS	58.65
11	*Cajanus cajan* (L.) Millsp.	Klwékoun (Fon, Goun); Otili (Nago)	H	S	AS	50.17
12	*Sorghum bicolor *(L.) Moench	Abôkoun (Fon, Mahi); Sooya (Wama)	H	S	AS	41.37
13	*Parkia biglobosa *(Jacq.) R. Br. ex Benth.	Ahwatin (Fon); Ayidan (Nago); Dooso (Dindi)	T	S, P	AS	40.68
14	*Dioscorea dumetorum* (Kunth) Pax	Léfé (Fon)	H	T	DS	38.27
15	*Sesamum radiatum* Thonn. ex Hornem.	Agbô (Fon, Mahi); Dossi (Bariba, Tchabè); Nonman (Wama, Yom); Toohoun (Berba), Touwadouanti (Ditamari), Touhoonôm (Lokpa)	H	L	RS	21.55
16	*Cyperus esculentus *L.	Fio (Yoruba, Fon); Gaasu (Dendi)	H	T	DS	18.79
17	*Blighia sapida* Koening	Lissètin (Fon); Ishin jije (Nago); Direbu (Bariba); Fisa (Dendi)	T	A, L	DS	17.06
18	*Irvingia gabonensis *(Aubry-Lecomte ex O'Rorke) Baill	Aslôtin (Fon)	T	Fr, N	DS	16.03
19	*Sphenostylis stenocarpa *(Hochst. ex A. Rich.) Harms	Sésé (Na)	H	S	AS	14.13
20	*Digitaria exilis *(Kippits) Stapf	Ipoaka (Ditamari); Péi (Wama); Poaji (Natimba); Ipoé (Bialli); Afiohoun (Lamba)	H	S	AS	13.1
21	*Adansonia digitata* L.	Kpassa (Fon, Mahi); Otché (Nago)	T	L, N, P	AS	10.68
22	*Justicia tenella *(Nees) T.	Atchélikéma (Ani), Kourôkountônu (Bariba, Boko, Peulh), Djagudjagu (Tchabè), Dimouniountchoro (Wama),	H	L	RS	9.65
23	*Ceratotheca sesamoides *Endl.	Agbô (Fon, Mahi); dowoungbaana (boko); Foyito (Dendi); Gblôgblô (Adja); Ebolo (Tchabè); Idjabô (Idasha); Kpééwori (Bariba); Siwadompéi (Ditamari); N'zoti (Kotokoli); Nor (Yom)	H	L	RS	9.31
24	*Bidens pilosa* L.	Djanwounkpi (Adja), Boboyo (Ani)	H	L	RS	8.96
25	*Cleome gynandra* L.	Akaya (Mahi), Axwouéssamboé (Watchi), Foubéyi (Dendi), Garcia (Bariba), Kiyépiéti (Natimba), Sabo (Adja, xwla), Sowounboyi (Kotokoli)	H	L	RS	8.44
26	*Acmella oleracea* (L.) R. K. Jansen	Oubouonou (Berba), Tipébouoti (Ditamari), Yoritampobou (Wama)	H	L	RS	7.06
27	*Sesamum indicum* L.	Saari (Wama, Natimba, Berba)	H	S	DS	4.17

TP: Type of plant; PU: part used; PA: Period of Availability; PR: percentage of respondents; H: Herb; T: Tree; Sh: Shrub; S: Seed; L: leaf; Fr: Fruit; T: Tuber, N: Nut, P: In-side fruit powder; A: Aril; DS: Dry season; AS: All season; RS: Rainy season.

**Table 4:** Reasons of neglect of the neglected and underutilised crop species and their relative importance according to farmers and the agricultural technicians.

Reasons	% of respondents	
	Farmers	Agricultural technicians
Lack of financial support for research	0	95.88
Lack of national promotion policy	80.73	89.41
Economically non profitable production	31.95	13.53
High cost of production	26.34	7.059
Lack of organised markets	98.04	65.88
Low yield	94.63	19.41
Painful production	49.02	0
Painful harvest	37.31	0
Susceptibility to high soil moisture	10.98	0
Susceptibility to low soil fertility	18.78	24.70
Susceptibility to pests and diseases	65.61	59.41
Susceptibility to weeds	15.61	0
Cultural believes	27.32	9.41
Lack of improved cultural practices and varieties	56.10	79.41
Ignorance of the species nutraceutical values	0	11.76

**Table 5:** Known nutritional values of some priority NUS in Benin.

Species	Nutritional values
*Cajanus cajan*	The levels of crude protein, crude fiber, K, Ca, P, and Mg reported on pigeon peas by Amarteifio et al. [30] were dry matter 86.6–88.0%, crude protein 19.0–21.7%, crude fat 1.2-1.3%, crude fiber 9.8–13.0%, and ash 3.9–4.3%. Minerals ranges (mg/100 g dry matter) were K 1845–1941, P 163–293, Ca 120–167, Mg 113–127, Na 11.3–12.0, Zn 7.2–8.2, Fe 2.5–4.7, and Cu 1.6–1.8. These data indicate that pigeon peas would positively contribute protein and could be valuable in the diet of the people in Benin.
*Ceratotheca sesamoides*	According to Fasakin [31] the leaves of *Ceratotheca sesamoides *used as vegetable contain low calories (soluble carbohydrates and fat) as well as high levels of protein (29.35–29.85%) and two of the elemental nutrients needed in large amounts by the human body, that is, calcium (2.60–2.62%) and phosphorus (0.25–0.27%). This vegetable is, therefore, a potent supplement to the starchy staple foods with which it is traditionally consumed and its consumption should be promoted, especially in families that can ill afford the prohibitive cost of animal protein.
*Citrullus lanatus *	The seeds consumed as vegetable is an excellent source of energy, protein, mineral salt, and vitamins. According to Das et al. [32], the composition of dried seed without shell per 100 g is water 5.1 g, energy 2340 kJ (557 kcal), protein 28.3 g, fat 47.4 g, carbohydrate 15.3 g, Ca 54 mg, P 755 mg, Fe 7.3 mg, thiamin 0.19 mg, riboflavin 0.15 mg, niacin 3.55 mg, and folate 58 *μ*g. The seed oil consists of glycosides of linoleic, oleic, palmitic, and stearic acids. It is a rich natural source of lycopene, a carotenoid of great interest because of its antioxidant capacity and potential health benefits [32].
*Cleome gynandra*	100 g edible portion of fresh leaves contain water 9.7 g, energy 34 kcal, protein 4.8 g, fat 0.4 g, carbohydrate 66.6 g, fibre 1.2 g, Ca 288 mg, P 111 mg, Fe 6.0 mg, and ascorbic acid 13 mg. It is a highly recommended meal for pregnant and lactating women [33].
*Corchorus olitorius*	*Corchorus Olitorius* leaves are rich sources of potassium, iron, copper, manganese and zinc as well as high energy values essential in human and animal nutrition. Idirs et al. [34] reported for 100 g of leaves: 18.38 g ash, 12.54 g crude protein, 11.99 g crude lipid, 19.56 g available carbohydrate, high energy value of 200.78 ± 3.54 kcal, potassium (2814.15 ± 8.08 mg) and magnesium (76.69 ± 0.13 mg), Na (54.56 mg), Ca (30.55 mg), P (6.68 mg), Cu (2.52 mg), Fe (19.53 mg), Mn (5.95 mg), and Zn (4.71 mg).
*Pennisetum glaucum*	The grain of pearl millet contains per 100 g edible portion: water 12.0 g, energy 1428 kJ (341 kcal), protein 10.4 g, fat 4.0 g, carbohydrate 71.6 g, fibre 1.9 g, Ca 22 mg, P 286 mg, Fe 20.7 mg, *β*-carotene traces, thiamin 0.30 mg, riboflavin 0.22 mg, niacin 1.7 mg, and ascorbic acid 3 mg. The content of essential amino acids per 100 g food is tryptophan 189 mg, lysine 332 mg, methionine 239 mg, phenylalanine 467 mg, threonine 374 mg, valine 535 mg, leucine 927 mg, and isoleucine 397 mg [35]. Compared to that of other millets, the protein of pearl millet is rich in tryptophan [35].
*Sesamum indicum *	Proximate composition and physicochemical analyses of the Sesamum seeds [36] used in Benin as frying vegetables showed that the seed contains 5.7% moisture, 20% crude protein, 3.7% ash, 3.2% crude fiber, 54% fat, and 13.4% carbohydrate. The seeds are good sources of minerals. Potassium (851.35 ± 3.44 mg/100 g) was the highest, followed in descending order by Phosphorus (647.25 ± 3.52 mg/100 g), Magnesium (579.53 ± 0.42 mg/100 g), Calcium (415.38 ± 3.14 mg/100 g), and Sodium (122.50 ± 4.21 mg/100 g). The physical properties of the oil extracts showed the state to be liquid at room temperature. The oil was found to contain high levels of unsaturated fatty acids, especially oleic (up to 38.84%), and linoleic (up to 46.26%). *Sesamum indicum* L. oil can be classified in the oleic-linoleic acid group. The dominant saturated acids were palmitic (up to 8.58%) and stearic (up to 5.44%). The oil extracts exhibited good physicochemical properties and could be useful as edible oils and for industrial applications.
*Sphenostylis stenocarpa*	African yam bean is a crop with food security potentials for Africa. The average composition of the seeds (whole grains) is as follows: protein 20.51%, fat 12.20%, carbohydrate 50.24%, ash 2.60%, fibre 6.00%, and moisture 8.36%. The seeds are rich in potassium and phosphorus (625.43 mg/100 g), and (206.35 mg/100 g) respectively [36, 37]. The content of crude protein in AYB seeds is lower than that in soybean, but the amino acid spectrum indicated that the level of most of the essential amino acids especially lysine, methionine, histidine, and isoleucine in AYB is higher than those in other legumes including soybean [36]. The seed is rich in minerals. The value of minerals per 100 g is: 4.2 g for P; 0.80 g for Ca; 1.7 g for Mg; 12.0 g for K; 0.06 g for Na; 46.2 mg for Mn; 44.0 mg for Zn; 63.0 mg for Fe 11.8 mg for Cu [36, 38].
*Vigna subterranea*	Following Amarteifio et al. [39] 100 g edible portion of dried seeds contain: water 10.3 g, energy 1537 kJ (367 kcal), protein 18.8 g, fat 6.2 g, carbohydrate 61.3 g, fibre 4.8 g, and ash 3.4 g. The ranges (mg/100 g dry matter) of the macrominerals in bambara groundnut are Ca 37–128, K 1545–2200, Mg 159–335, Na 16–25, and P 313–563. For the microminerals (ppm): Cu 3.0–13.2, Fe 23.0–150 and Zn 13.9–77.0, *β*-carotene 10 *μ*g, thiamin 0.47 mg, riboflavin 0.14 mg, niacin 1.8 mg and ascorbic acid traces. The content of essential amino acids per 100 g food is tryptophan 192 mg, lysine 1141 mg, methionine 312 mg, phenylalanine 991 mg, threonine 617 mg, valine 937 mg, leucine 1385 mg, and isoleucine 776 mg [40]. This legume is a good source of Ca, K, Mg, P, and Fe and its concentration in minerals indicates that it could be useful in the diets of consumers.

**Table 6:** Market value of the priority NUS at Glazoué market as indicated by the market's women association.

Species	Price per Kg in US $	
	Minimum	Maximum
*Bidens pilosa*	0.375	0.5
*Blighia sapida*	2	2.5
*Cajanus cajan*	0.875	1
*Ceratotheca sesamoides*	1.25	1.5
*Citrullus lanatus *	2	2.25
*Cleome gynandra*	0.5	0.75
*Corchorus olitorius*	1.5	3
*Crassocephalum crepidiondes*	1.25	1.75
*Crassocephalum rubens*	1.25	1.75
*Cucumeropsis mannii*	2.25	2.5
*Cyperus esculentus *	2	2.5
*Dioscorea dumetorum *	2	2.25
*Ipomea batatas*	1.25	1.5
*Launaea taraxacifolia*	0.625	1.125
*Macrotyloma geocarpum*	2.25	3.75
*Pennisetum glaucum*	0.75	1
*Sesamum indicum *	1.75	2.25
*Sphenostylis stenocarpa*	0.625	0.75
*Vigna subterranea*	1.5	2

**Table 7:** Contribution of both men and women to the production and marketing of the priority neglected and underutilized crops species in Benin.

Species	North				Centre and south			
	Production		Marketing		Production		Marketing	
	Men	Women	Men	Women	Men	Women	Men	Women
*Bidens pilosa*	+	+++	+	+++	+	+++	+	+++
*Blighia sapida*	+++	+	+	+++	+++	+	+	+++
*Cajanus cajan*	+	+++	+	+++	+++	+++	+	+++
*Ceratotheca sesamoides*	+	+++	+	+++	+	+++	+	+++
*Citrullus lanatus *	+++	+++	+	+++	+++	+++	+	+++
*Cleome gynandra*	+	+++	+	+++	+	+++	+	+++
*Corchorus olitorius*	+	+++	+	+++	+	+++	+	+++
*Crassocephalum rubens*	+	+++	+	+++	+	+++	+	+++
*Crassocephalum crepidioides*	+	+++	+	+++	+	+++	+	+++
*Cucumeropsis mannii*	+++	+++	+	+++	+++	+++	+	+++
*Cyperus esculentus*	+++	+++	+	+++	nc	nc	nc	nc
*Dioscorea dumetorum *	+++	+	+	+++	+++	+	+	+++
*Ipomea batatas *	+++	+++	+	+++	+++	+++	+	+++
*Launaea taraxacifolia*	+	+++	+	+++	+	+++	+	+++
*Macrotyloma geocarpum*	+	+++	+	+++	+++	+++	+	+++
*Pennisetum glaucum*	+++	+++	+++	+++	+++	+++	+	+++
*Sesamum indicum *	+++	+++	+++	+++	nc	nc	nc	nc
*Sphenostylis stenocarpa*	+++	+	+++	+++	+++	+++	+	+++
*Vigna subterranea*	+	+++	+	+++	+++	+++	+	+++

**Table 8:** Research needs on the priority neglected and underutilised crop species of Benin.

Species	Research topics											
	ET	PC	DO	AG	IA	PD	AE	SQ	BC	PH	VC	GC
*Bidens pilosa*	x	x	x	x	x	x	x		x		x	x
*Blighia sapida*		x				x	x		x	x	x	x
*Cajanus cajan*	x	x		x	x	x	x				x	x
*Ceratotheca sesamoides*		x	x		x	x	x	x			x	x
*Citrullus lanatus *				x	x	x	x			x	x	x
*Cleome gynandra*	x	x	x	x	x	x	x				x	x
*Corchorus olitorius*	x	x		x	x	x	x				x	x
*Crassocephalum crepidioides*	x	x	x	x	x	x	x				x	x
*Crassocephalum rubens*	x	x	x	x	x	x	x				x	x
*Cucumeropsis mannii*				x	x	x	x			x	x	x
*Cyperus esculentus *	x	x		x	x	x	x	x	x	x	x	x
*Dioscorea dumetorum *	x	x		x	x	x	x		x	x	x	x
*Ipomea batatas*	x	x		x		x	x		x	x	x	x
*Launaea taraxacifolia*	x	x	x	x	x	x	x	x	x		x	x
*Macrotyloma geocarpum*	x	x		x	x	x	x			x	x	x
*Pennisetum glaucum*	x	x		x		x	x			x	x	x
*Sesamum indicum*	x	x		x	x	x	x			x	x	x
*Sphenostylis stenocarpa*	x	x		x	x	x	x				x	x
*Vigna subterranea*	x	x		x	x	x	x				x	x

ET: ethnobotanical investigation and documentation of the indigenous knowledge; PC: identification and prioritisation of the production constraints; DO: domestication; AG: agromorphological characterisation and genetic diversity analysis; IA: improvement of the agricultural practices; PD: documentation of the pests and diseases; AE: agronomic (yield, biotic and abiotic stresses) evaluation; SQ: assessment of the seeds quality and conservation; BC: analysis of the biochemical composition and assessment of the nutritional values; PH: improvement of postharvest conservation and processing technologies; VC: study of the value chains and assessment of the contribution to household income; GC: germplasm collection and conservation.
